# Does Retroperitoneal vNOTES Sentinel Lymph Node Mapping Represent a Feasible Staging Option in Presumed Early-Stage Endometrial Cancer?

**DOI:** 10.3390/medicina62010043

**Published:** 2025-12-25

**Authors:** Behzat Can, Kevser Arkan, Ali Deniz Erkmen, Sedat Akgol

**Affiliations:** Department Obstetrics and Gynecology, Division of Gynecologic Oncology, Diyarbakir Gazi Yasargil Research and Training Hospital, 21010 Diyarbakir, Turkey; kevser.toprak1989@gmail.com (K.A.); erkmendnz@gmail.com (A.D.E.); drsedatakgol@gmail.com (S.A.)

**Keywords:** vaginal natural orifice transluminal endoscopic surgery (vNOTES), endometrial cancer, laparoscopy, staging surgery, retroperitoneal dissection

## Abstract

*Background and Objectives*: Sentinel lymph node (SLN) mapping is an established alternative to systematic lymphadenectomy for early-stage endometrial cancer (EC). While retroperitoneal vNOTES affords direct access to pelvic nodes without abdominal incisions, data regarding its oncologic validity remain sparse. This study evaluates the SLN detection rates, perioperative outcomes, and 12-month oncologic outcomes oncologic results of retroperitoneal vNOTES mapping in presumed early-stage EC. *Materials and Methods*: This single-center retrospective cohort study analyzed consecutive patients undergoing retroperitoneal vNOTES staging (hysterectomy, BSO, and SLN mapping) for presumed EC between February 2023 and January 2024. Eligible patients had radiologically uterine-confined disease and were candidates for transvaginal surgery. Following cervical methylene blue injection, SLN mapping was executed via the retroperitoneal vNOTES route. Mapped and suspicious nodes were excised, with side-specific lymphadenectomy performed for failed mapping per algorithm. While perioperative outcomes were assessed for the full cohort, oncologic analyses (FIGO 2023 staging, nodal metastasis) were restricted to patients with confirmed carcinoma. *Results:* Of 98 patients (median age 54; BMI 31 kg/m^2^), final pathology confirmed carcinoma in 78 (73 endometrioid, 5 serous) and EIN in 20. Bilateral SLN mapping succeeded in 87.8% (86/98), necessitating side-specific lymphadenectomy in the remaining 12.2%. The obturator fossa was the predominant nodal basin (43.9%). Within the carcinoma cohort (*n* = 78), 57.7% were Grade 1 and 74.4% FIGO Stage I. Nodal metastases (FIGO IIIC1) were identified in 12.8% (10/78), all prompting adjuvant therapy. At a median follow-up of 12 months, no disease recurrences were observed. The complication rate was 6.1% (5.1% Clavien–Dindo ≥ III), with no conversions required. At 12-month follow-up, no recurrences were detected, though the absence of systematic lymphadenectomy precluded formal sensitivity analysis. *Conclusions*: Retroperitoneal vNOTES represents a feasible and safe strategy for SLN mapping in early-stage EC, demonstrating high bilateral detection with minimal morbidity. However, reliance on methylene blue and limited follow-up necessitate caution. Broader implementation requires validation through prospective, comparative trials utilizing indocyanine green and long-term oncologic surveillance.

## 1. Introduction

Endometrial cancer (EC) remains the most prevalent gynecologic malignancy in developed nations [1]. While the majority of patients present with clinically uterine-confined disease, accurate lymph node (LN) assessment is pivotal for staging, prognostication, and tailoring adjuvant therapy [1,2].

Historically, systematic lymphadenectomy was the standard for nodal evaluation. However, given its lack of survival benefit in early-stage disease and association with significant morbidity, it has largely been replaced by sentinel lymph node (SLN) mapping [2,3,4,5]. Major guidelines now endorse adherence to validated algorithms that mandate the excision of mapped nodes and side-specific lymphadenectomy in cases of mapping failure [2,3,4,6].

Concurrently, minimally invasive surgery has become the preferred approach, offering equivalent oncologic safety to laparotomy with superior recovery profiles [2,7]. The integration of SLN mapping into these protocols represents the optimal balance between accurate staging and minimal surgical impact [3,4,5].

Vaginal natural orifice transluminal endoscopic surgery (vNOTES) represents a distinct evolution in minimally invasive gynecology. Initially validated for benign indications, vNOTES offers reduced pain, shorter hospitalization, and improved cosmesis compared to laparoscopy [8,9,10,11,12]. Consequently, the technique has expanded to gynecologic oncology, including full staging procedures [10,13,14,15]. Specifically, the retroperitoneal vNOTES approach facilitates SLN mapping by providing direct access to pelvic basins and early visualization of lymphatic channels with minimal bowel manipulation [15,16,17].

Despite preliminary success, data on retroperitoneal vNOTES SLN mapping remain limited to select centers predominantly utilizing indocyanine green (ICG). Evidence regarding its feasibility in public tertiary settings using methylene blue is particularly scarce. Validating this approach under such real-world conditions is therefore clinically critical.

This retrospective single-center study evaluates retroperitoneal vNOTES SLN mapping during staging for presumed early-stage EC. Primary endpoints were SLN detection rates and anatomical distribution. Secondary endpoints included perioperative morbidity and oncologic outcomes at 12-month follow-up. Perioperative data were analyzed for the entire cohort, whereas oncologic metrics were restricted to patients with confirmed carcinoma.

## 2. Materials and Methods

### 2.1. Study Design and Setting

This single-center retrospective cohort study was conducted at Diyarbakir Gazi Yasargil Training and Research Hospital, a high-volume tertiary gynecologic oncology referral center in Turkey. Following structured training and institutional credentialing, retroperitoneal vNOTES was established at our department as a standard minimally invasive modality for selected gynecologic malignancies. All procedures analyzed herein were performed as part of routine clinical care for patients with confirmed or suspected endometrial cancer. The decision to utilize the vNOTES approach was determined by clinical indication, anatomical suitability, and patient counseling rather than for data collection purposes.

### 2.2. Ethical Approval

This study adhered to the principles of the Declaration of Helsinki and received approval from the Institutional Review Board of the University of Health Sciences, Diyarbakir Gazi Yasargil Training and Research Hospital (Approval No. 169; 13 September 2024). All patients provided written informed consent for the surgical procedure, including the specific minimally invasive approach and nodal staging, as well as for the use of anonymized clinical data for scientific publication.

### 2.3. Patient Selection

Consecutive women undergoing retroperitoneal vNOTES hysterectomy, bilateral salpingo-oophorectomy, and sentinel lymph node (SLN) mapping for presumed endometrial cancer between February 2023 and January 2024 were identified from a prospective institutional database. Inclusion required a preoperative histologic diagnosis of EC or endometrial intraepithelial neoplasia (EIN) and clinically uterine-confined disease based on contrast-enhanced CT, MRI, or 18F-FDG PET/CT. Surgical candidates were selected based on an ECOG performance status of 0–1, adequate transvaginal access, and a uterine size consistent with ≤12 gestational weeks.

Exclusion criteria encompassed prior pelvic/paraaortic lymphadenectomy or radiotherapy, severe endometriosis, fixed uteri, BMI > 40 kg/m^2^, and methylene blue contraindications. Crucially, patients with radiologically suspicious lymphadenopathy (defined as short axis diameter > 10 mm, loss of fatty hilum, or FDG avidity) were excluded, as these cases mandated comprehensive lymphadenectomy via conventional laparoscopic or open approaches. Following multidisciplinary review and counseling regarding the vNOTES technique, consenting patients were enrolled.

To reflect real-world applicability, perioperative feasibility outcomes, including operative time, complications, and hospital stay, were analyzed for the entire cohort on an intention-to-treat basis. Conversely, oncologic endpoints such as FIGO staging, nodal metastasis rates, and recurrence were restricted to patients with a final pathologic diagnosis of carcinoma, explicitly excluding EIN cases from survival analyses.

### 2.4. Preoperative Evaluation

Preoperative assessment comprised standard physical examination, laboratory panels, and transvaginal ultrasonography. Cross-sectional imaging with MRI or contrast-enhanced CT was mandatory for all patients. 18F-FDG PET/CT was utilized selectively for high-risk or equivocal cases. Clinical stage I disease was defined by the radiologic absence of adnexal pathology, lymphadenopathy, peritoneal carcinomatosis, or distant metastasis.

### 2.5. Surgical Procedure

All procedures were executed by a dedicated gynecologic oncology team proficient in vNOTES and SLN mapping. While trainees participated under direct supervision, senior surgeons performed all critical steps, including retroperitoneal dissection, SLN excision, and hysterectomy, in adherence to a standardized institutional protocol.

### 2.6. Injection and Preparation

Under general anesthesia and dorsal lithotomy positioning, standard antibiotic prophylaxis was administered. To maintain oncologic integrity, uterine manipulators were strictly avoided. Following vaginal preparation, a total of 4 mL of methylene blue (40 mg) was injected intracervically at the 3 and 9 o’clock positions. At each site, 1 mL was injected superficially (1–3 mm) and 1 mL deeply (10–15 mm). A 15 min interval was observed post-injection to ensure adequate lymphatic migration prior to dissection.

### 2.7. Retroperitoneal vNOTES Access and SLN Mapping

The retroperitoneal approach followed the concept originally described by Baekelandt, as further refined in our institutional experience [13,14]. Following cervical injection, the cervix was tractioned contralaterally to expose the paracervical space and safeguard the bladder. A 2 cm vertical mucosal incision was created at the 3–4 o’clock position (right side). Dissection advanced along the paravesical plane toward the internal obturator muscle and obturator foramen.

**Figure 1:**
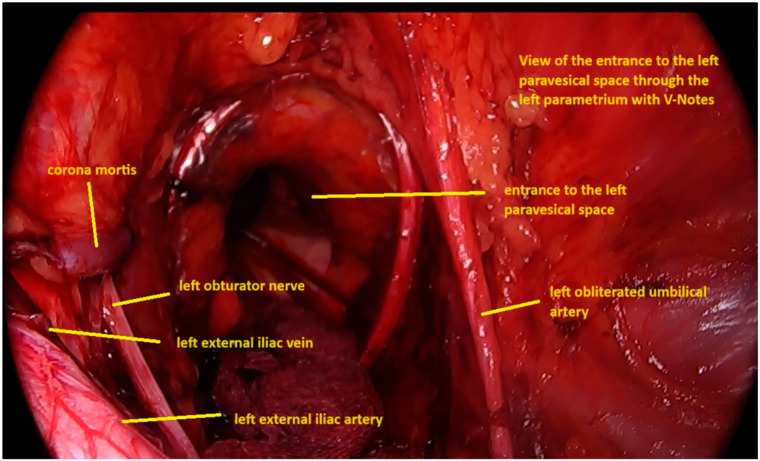
Intraoperative view of the entrance to the left paravesical space through the left parametrium with vNOTES.

Progression continued in a caudal to cranial vector until the bifurcation of the external and internal iliac veins was palpated, confirming entry into the correct retroperitoneal plane and the obturator fossa. This direct access facilitated the identification of the obturator nerve, iliac vessels, and pelvic sidewall landmarks (Figure 1). Upon adequate development of the space, a 7 cm GelPoint V-Path transvaginal access platform (Applied Medical, Rancho Santa Margarita, CA, USA) was deployed.

Instrumentation included a central 10 mm port for a 30° endoscope and two 5 mm lateral ports for a bipolar grasper and advanced sealing device. Pneumoretroperitoneum was maintained at 12 mmHg, ensuring optimal visualization while preserving retroperitoneal integrity. Systematic dissection proceeded medially to laterally; the ureter, umbilical artery, and iliac vessels were skeletonized as required. The retroperitoneal tissue was inspected for afferent lymphatic channels, with the SLN defined as the first blue-stained node in each hemipelvis.

**Figure 2:**
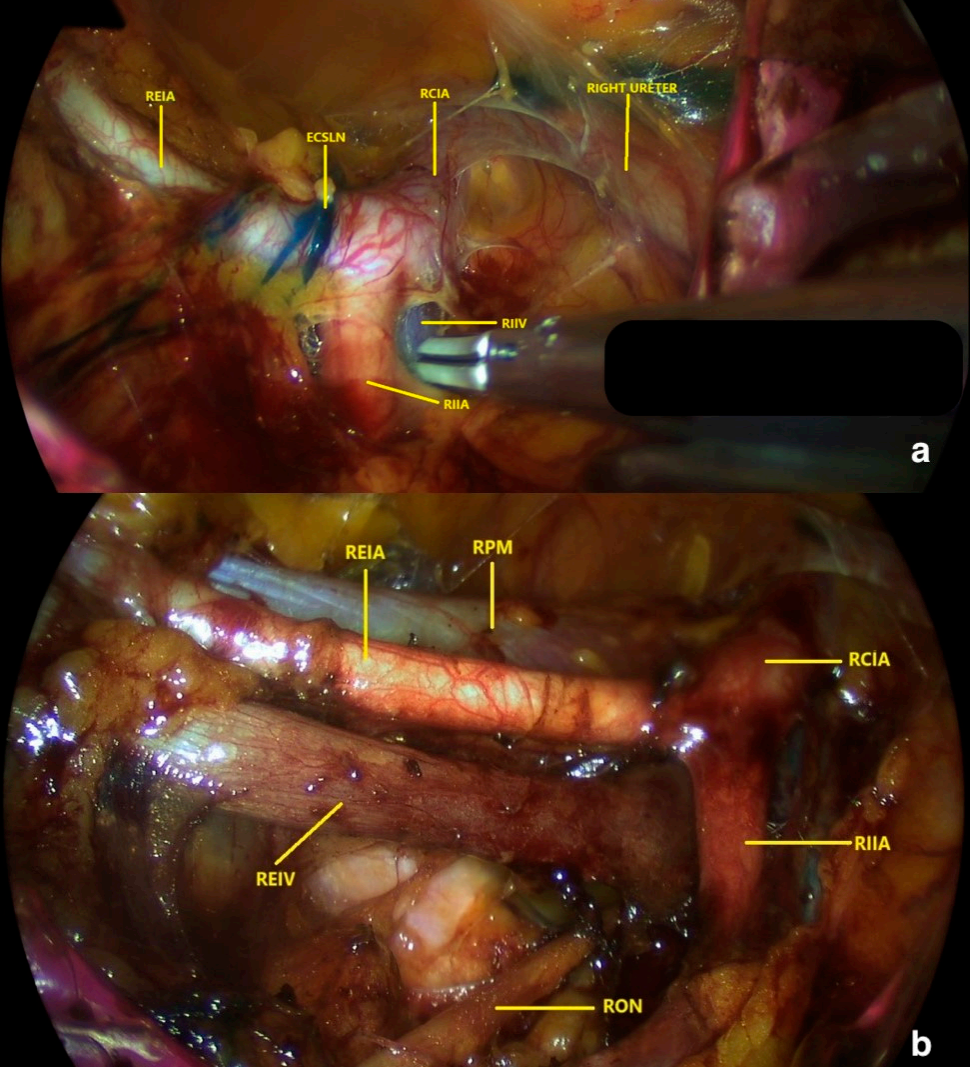
(**a**) Anatomical view of the right pelvic paravesical area via retroperitoneal transvaginal approach with vNOTES and (**b**) anatomical view of the right pelvic paravesical area following lymph node dissection via retroperitoneal transvaginal approach with vNOTES. (ECSLN: efferent channels of the sentinel lymph node; RCIA: right common iliac artery; REIA: right external iliac artery; REIV: right external iliac vein; RIIA: right internal iliac artery; RIIV: right internal iliac vein; RON: right obturator nerve; RPM: right psoas muscle).

Identified SLNs and any macroscopically suspicious nodes were excised en bloc with surrounding adipose tissue to maintain capsular integrity. Specimens were retrieved transvaginally via the GelPoint platform to prevent seeding. Figure 2 illustrates the right obturator fossa before and after dissection. Following completion of the right hemipelvis, the identical procedure was executed on the left side.

Nodal management adhered to a predefined algorithm consistent with contemporary guidelines [2,3,6,8,9,18]:

Bilateral Mapping: Excision of mapped SLNs and any suspicious nodes only.

Unilateral Mapping: SLN excision on the mapped side, coupled with side-specific lymphadenectomy (external/internal iliac and obturator basins) on the unmapped side.

Mapping Failure: Bilateral pelvic lymphadenectomy performed at the surgeon’s discretion. The sentinel lymph node algorithm applied in this study is summarized in Supplementary Table S1.

Paraaortic nodal assessment was not routine and was reserved for cases with suspicious intraoperative findings, enlarged nodes on preoperative imaging, or aberrant drainage patterns.

### 2.8. vNOTES-Assisted Hysterectomy and BSO

Nodal specimens underwent formalin fixation and serial sectioning by dedicated gynecologic pathologists. Routine evaluation utilized hematoxylin-eosin (H&E) staining, with reflex immunohistochemistry for cytokeratin applied selectively for equivocal morphology or suspected micrometastasis.

Tumors were classified per World Health Organization (WHO) criteria and staged according to the 2023 FIGO system. Staging parameters, lymphovascular space invasion (LVSI), and nodal status were reported exclusively for confirmed carcinoma. Conversely, EIN cases were categorized as preinvasive lesions without FIGO stage assignment.

For node-positive patients, metastatic burden was stratified as macrometastasis, micrometastasis, or isolated tumor cells (ITCs). Adjuvant management was adjudicated by a multidisciplinary tumor board in adherence to NCCN and ESGO/ESTRO/ESP consensus guidelines [2,8,18].

Supplemental File S1 provides a video demonstration of the retroperitoneal access, SLN mapping, and hysterectomy technique.

### 2.9. Histopathological Evaluation and Staging

All excised SLNs and non-SLNs were fixed in formalin and processed by dedicated gynecologic pathologists. Nodes were serially sectioned and evaluated with hematoxylin–eosin staining according to institutional protocols. When morphologic findings were equivocal or micrometastasis was suspected, additional levels and immunohistochemistry for cytokeratin were performed at the pathologist’s discretion.

Endometrial tumors were classified according to World Health Organization (WHO) criteria and graded as grade 1, 2, or 3. EC cases were staged using the 2023 FIGO staging system. FIGO staging, lymphovascular space invasion (LVSI), SLN metastasis rates, and nodal staging outcomes were reported only for patients with a final diagnosis of carcinoma. EIN cases were reported separately as preinvasive lesions and were not assigned a FIGO stage.

For SLN-positive patients, the presence of macrometastasis, micrometastasis, or isolated tumor cells was recorded when available, and adjuvant treatment was determined in a multidisciplinary tumor board according to NCCN and ESGO/ESTRO/ESP recommendations [2,8,18].

### 2.10. Postoperative Care and Follow-Up

Postoperative management adhered to institutional enhanced recovery pathways. Early recovery metrics included mobilization latency (hours), length of hospital stay (days), and pain intensity assessed via the Visual Analogue Scale (VAS) at 6 and 24 h post-surgery.

Perioperative complications occurring within 30 days were graded according to the Clavien–Dindo classification. Morbidity was categorized by system: urinary tract, vascular, neurologic, vaginal cuff-related, or other.

Surveillance entailed an initial clinical evaluation at 4–6 weeks, followed by 3–6 month intervals during the first postoperative year. Visits comprised symptom review and pelvic examination, with adjuvant imaging (US, CT, or MRI) dictated by clinical suspicion. Oncologic endpoints assessed at 12 months encompassed survival status and the site (local, nodal, or distant) and timing of any recurrence.

### 2.11. Outcomes and Statistical Analysis

The primary endpoint was the bilateral SLN detection rate utilizing retroperitoneal vNOTES with cervical methylene blue injection. Secondary endpoints encompassed the overall detection rate, unilateral mapping frequency, and nodal distribution. Clinical outcomes included perioperative metrics (operative time, estimated blood loss, mobilization latency, hospital stay, VAS pain scores) and complication rates graded via the Clavien–Dindo classification. Pathologic endpoints comprised histologic subtype, grade, LVSI, tumor size, FIGO 2023 staging, and nodal metastasis rates. Oncologic efficacy was assessed based on recurrence patterns and survival status at 12 months.

Data extraction adhered to a standardized protocol with independent cross-verification by two investigators to minimize transcription errors. Statistical analyses were conducted using IBM SPSS Statistics for Windows, version 25.0 (IBM Corp., Armonk, NY, USA) Continuous variables are reported as mean ± standard deviation or median (range) contingent on normal distribution assessed via Kolmogorov–Smirnov and Shapiro–Wilk tests. Categorical variables are expressed as frequencies and percentages.

Given the retrospective single-center design and sample size, analyses were primarily descriptive. No formal power analysis or multivariable modeling was undertaken. Calculation of sensitivity and false-negative rates was precluded by the absence of systematic lymphadenectomy in unmapped hemipelvises and the limited follow-up duration.

## 3. Results

The study cohort comprised 98 patients with a median age of 54 years and a median BMI of 31 kg/m^2^. Obstetric history was predominantly multiparous (89.8%), while 7.1% were nulliparous. Prior abdominal surgery was noted in 4.1% of cases (Table 1).

### 3.1. Perioperative Outcomes

Detailed perioperative metrics are provided in Table 1. The median operative time was 109.5 min, accompanied by a median estimated blood loss of 60 mL. Postoperative hemoglobin levels remained stable (median 11.5 g/dL). Recovery was rapid, evidenced by a median mobilization latency of 5 h and a hospital stay of 2 days. Postoperative pain was minimal, with median VAS scores declining to 1 by the 24th postoperative hour.

### 3.2. SLN Detection and Lymph Node Dissection

Retroperitoneal vNOTES mapping demonstrated high technical feasibility. Bilateral SLN detection was achieved in 86 patients (87.8%). The remaining 12 patients (12.2%) exhibited unilateral mapping (8 right-only, 4 left-only), necessitating side-specific lymphadenectomy on the unmapped hemipelvis. The median SLN yield was 2 on the right and 3 on the left. In cases requiring therapeutic lymphadenectomy due to mapping failure, the mean non-SLN yield was 9.6 on the right and 9.7 on the left. Anatomically, the obturator fossa constituted the predominant sentinel basin (43.9%), followed by the internal (31.6%) and external iliac (24.5%) regions (Table 2).

### 3.3. Histopathological Findings and Staging

Final histopathology confirmed carcinoma in 78 patients (74.5% endometrioid, 5.1% serous) and EIN in 20 (20.4%). Consequently, oncologic staging and outcome analyses were restricted to the 78 carcinoma cases. Within this cohort, low-grade (Grade 1) histology predominated (57.7%), and lymphovascular space invasion (LVSI) was identified in 17.9%. The median tumor diameter was 2.5 cm (Table 2).

Based on the 2023 FIGO classification, the majority (74.4%) presented with Stage I disease. Stage II and Stage III were confirmed in 11.5% and 14.1% of patients, respectively. A granular breakdown of specific substages is provided in Table 2.

### 3.4. SLN Metastases and Adjuvant Treatment

Nodal metastases were identified in 10 patients, representing 12.8% of the carcinoma cohort. All node-positive cases were consequently upstaged to FIGO IIIC1. Importantly, no additional metastatic non-sentinel lymph nodes were identified in patients who underwent side-specific lymphadenectomy due to unilateral mapping failure, supporting adherence to the sentinel lymph node algorithm.

Clinicopathologic characteristics and adjuvant protocols for node-positive patients are detailed in Table 3. Chemotherapy was administered in 90% (9/10) of these cases, with pelvic external beam radiotherapy (EBRT) and vaginal brachytherapy (VBT) utilized in 70% and 30%, respectively. Selective paraaortic assessment performed in two cases with intraoperative suspicion proved negative for metastasis.

### 3.5. Complications and 12 Month Oncologic Outcomes

The overall complication rate was 6.1% (6/98). Major morbidity (Clavien–Dindo ≥ III) occurred in 5.1%, comprising three Grade IIIa and two Grade IIIb events. Specific adverse events included bladder injury (n = 2), vaginal cuff hematoma (n = 2), and isolated instances of external iliac vein and obturator nerve injury. Both bladder defects were repaired via the vaginal route, while one hematoma required drainage. Crucially, all complications were managed through minimally invasive or vaginal approaches, with zero conversions to laparotomy or conventional laparoscopy.

At the 12-month surveillance mark, no local, nodal, or distant recurrences were detected within the carcinoma cohort. Specifically, no nodal failures occurred in hemipelvises classified as SLN negative. However, given the absence of systematic lymphadenectomy and the limited observational period, a definitive false-negative rate could not be derived.

## 4. Discussion

This study demonstrates that retroperitoneal vNOTES staging is a feasible and safe strategy for presumed early-stage endometrial cancer (EC). In a cohort of 98 patients, we achieved a high bilateral SLN detection rate (87.8%) with minimal perioperative morbidity. Among the 78 patients with confirmed carcinoma, nodal metastases were identified in 12.8%, all of whom were successfully upstaged to FIGO IIIC. While the absence of recurrences at the 12-month mark is encouraging, definitive assertions regarding long-term oncologic safety are naturally tempered by the limited follow-up duration.

From a clinical decision-making perspective, retroperitoneal vNOTES should be viewed as a complementary minimally invasive approach rather than a universal substitute for laparoscopy. This technique may offer particular advantages in carefully selected patients with uterine confined disease, favorable vaginal access, and limited prior pelvic surgery, where direct retroperitoneal access allows early identification of pelvic lymphatic pathways with minimal bowel manipulation. Conversely, transperitoneal or extraperitoneal laparoscopic approaches may remain preferable in patients with extensive adhesions, suspected extrauterine disease, or when comprehensive abdominal exploration is anticipated.

Our SLN detection metrics align with established benchmarks in EC management [4,8]. Achieving an 87.8% bilateral detection rate using methylene blue via the retroperitoneal route is notable. While indocyanine green (ICG) is consistently associated with higher bilateral detection rates in contemporary series, the results observed in our cohort exceed those reported in most traditional blue dye studies and demonstrate that acceptable detection rates can be achieved without fluorescence imaging in experienced hands. Furthermore, the anatomical distribution of SLNs, predominantly within the obturator and iliac basins, mirrors patterns reported for conventional laparoscopic approaches, supporting the anatomical fidelity of the retroperitoneal vNOTES technique. The median retrieval of 2–3 SLNs per hemipelvis further aligns with established anatomic and clinical standards [5,19].

A pivotal strength of this analysis lies in its strict adherence to guideline-endorsed SLN algorithms. By mandating the excision of mapped nodes and performing systematic side-specific lymphadenectomy upon mapping failure, this protocol minimizes the risk of understaging [2,6,9]. Within the carcinoma cohort, nodal metastases were identified in 12.8% of cases. Notably, no additional metastases were found during completion lymphadenectomy for unilateral mapping. However, the absence of systematic lymphadenectomy across all hemipelvises precludes the calculation of a formal false negative rate, necessitating cautious interpretation regarding diagnostic accuracy.

Perioperative metrics in this cohort parallel established standards for minimally invasive staging. The acceptable operative times and minimal blood loss, coupled with early mobilization and a median two-day discharge, underscore the recovery benefits of the retroperitoneal vNOTES technique. Furthermore, the low postoperative pain scores likely reflect the avoidance of abdominal incisions. The 6.1% overall complication rate, including a 5.1% rate of major morbidity (Clavien–Dindo ≥ III), aligns with advanced laparoscopic and robotic series [10]. While all adverse events were managed via minimally invasive or conservative measures, the occurrence of specific vascular and nerve injuries confirms that retroperitoneal dissection carries inherent risks. Consequently, this approach demands proficiency in pelvic retroperitoneal anatomy and vNOTES ergonomics [11,12,15].

This study underscores the necessity of rigorous patient selection when implementing novel surgical techniques in public healthcare. We avoided indiscriminate application by restricting the approach to candidates with clinically uterine confined disease, appropriate vaginal access, and adequate performance status. Surgical decision-making relied on multidisciplinary consensus and patient counseling. Crucially, all procedural steps aligned with standard oncologic staging rather than experimental data collection, ensuring that innovation did not compromise patient safety or responsible resource allocation.

Regarding tracer selection, while contemporary guidelines prioritize indocyanine green (ICG) for its superior detection profiles, our exclusive reliance on methylene blue was initially dictated by local resource constraints. Although fluorescence imaging capabilities were acquired during the study period, we elected to maintain the methylene blue protocol to preserve series homogeneity. This decision also served to validate the feasibility of the technique for institutions where access to advanced optical technology remains restricted. Despite this technical limitation, the high bilateral detection rates and clinically significant nodal yields suggest that retroperitoneal vNOTES remains effective with traditional dyes in experienced hands. Future comparative studies are essential to quantify the incremental benefit of fluorescence-guided vNOTES mapping [4,8,18].

The inclusion of EIN cases in feasibility analyses reflects the frequent discordance between preoperative sampling and final pathology in daily practice. While we excluded these patients from staging analyses to maintain oncologic clarity, their retention underscores the real-world applicability of the surgical workflow. Similarly, the inclusion of a small subset of serous carcinomas warrants cautious interpretation. Although SLN mapping is increasingly validated for high-risk histologies [2,8,18], our study was not powered to draw histology-specific conclusions.

Several limitations warrant mention. Firstly, the retrospective single-arm design lacks a control group, precluding direct comparison with conventional laparoscopy or robotics [20,21,22]. Secondly, strict selection criteria, including the exclusion of patients with a BMI > 40 kg/m^2^, were applied to ensure procedural safety during the early implementation of the retroperitoneal vNOTES technique. While this approach enhanced internal validity, it inevitably limits the generalizability of our findings to morbidly obese populations, which represent a clinically relevant subgroup in endometrial cancer. Third, the descriptive nature of the analysis and the 12-month follow-up period limit definitive assertions regarding long-term survival, as recurrences frequently manifest later in the disease course [2,11]. Finally, the absence of systematic pelvic and paraaortic lymphadenectomy in all patients precludes the calculation of formal sensitivity and false negative rates, which should be addressed in future prospective comparative studies.

## 5. Conclusions

Retroperitoneal vNOTES SLN mapping represents a feasible and safe staging strategy for carefully selected patients with presumed early-stage endometrial cancer. Our findings demonstrate that high bilateral detection rates and favorable perioperative outcomes are achievable without conversion, even when utilizing methylene blue. While these preliminary results are encouraging, establishing long-term oncologic equivalence mandates validation through larger prospective trials. Future research should prioritize indocyanine green protocols and direct comparison with standard laparoscopic or robotic approaches to define the precise role of this technique in routine cancer care [13,14,16,23].

## Figures and Tables

**Table 1:** Baseline clinical characteristics and perioperative outcomes of the study cohort (N = 98).

Variable	Median (Range) or n (%)
**Clinical characteristics**	
Age (years)	54 (34–78)
BMI (kg/m^2^)	31 (25–38)
Nulliparity	7 (7.1)
Previous vaginal delivery	88 (89.8)
Previous cesarean section	20 (20.4)
Previous abdominal surgery	4 (4.1)
**Perioperative outcomes**	
Preoperative hemoglobin (g/dL)	12.7 (10.0–15.7)
Preoperative hematocrit (%)	38.6 (30.8–49.0)
Postoperative hemoglobin (g/dL)	11.5 (7.3–13.8)
Postoperative hematocrit (%)	35.3 (23.2–44.2)
Duration of surgery (min)	109.5 (82–160)
Estimated blood loss (mL)	60 (30–450)
VAS score at 6 h	3 (1–4)
VAS score at 24 h	1 (1–4)
Time to mobilization (h)	5 (4–7)
Length of hospital stay (days)	2 (1–6)

BMI: body mass index; VAS: visual analog scale.

**Table 2:** SLN detection, anatomical distribution, and histopathological results.

Variable	n (%) or Median (Range)
**SLN mapping outcomes (N = 98)**	
SLN identified only on right side	8 (8.2)
SLN identified only on left side	4 (4.1)
SLNs identified bilaterally	86 (87.8)
**Number of lymph nodes excised**	
Right SLNs, per patient	2 (0–9)
Left SLNs, per patient	3 (0–9)
Right non-SLNs in contralateral lymphadenectomy (n = 12)	9.6 (9–10) *
Left non-SLNs in contralateral lymphadenectomy (n = 12)	9.7 (7–12) *
**SLN location (dominant basin per patient)**	
Obturator region	43 (43.9)
Internal iliac region	31 (31.6)
External iliac region	24 (24.5)
**Final histopathology (N = 98)**	
Endometrioid carcinoma	73 (74.5)
Serous carcinoma	5 (5.1)
Endometrial intraepithelial neoplasia (EIN)	20 (20.4)
**Tumor grade (carcinoma only, N = 78)**	
Grade 1	45 (57.7)
Grade 2	24 (30.8)
Grade 3	9 (11.5)
**Additional pathological features (carcinoma only)**	
LVSI present	14 (17.9)
Tumor diameter (cm)	2.5 (0–8)
Patients with SLN metastasis	10 (12.8) **
**FIGO 2023 stage (carcinoma only, N = 78)**	
Stage IA1	18 (23.1)
Stage IA2	26 (33.3)
Stage IA3	4 (5.1)
Stage IB	10 (12.8)
Stage IIA	3 (3.8)
Stage IIB	2 (2.6)
Stage IIC	4 (5.1)
Stage IIIA2	1 (1.3)
Stage IIIC1	10 (12.8)

SLN: sentinel lymph node; EIN: endometrial intraepithelial neoplasia; LVSI: lymphovascular space invasion; FIGO: International Federation of Gynecology and Obstetrics (2023 staging). * Mean (range). ** Calculated based on the 78 patients with final histopathology of carcinoma (excluding 20 EIN cases).

**Table 3:** Clinicopathologic and adjuvant treatment characteristics of SLN-positive patients (n = 10).

Patient	Age (Years)	Histology	Grade	LVSI	Tumor Size (cm)	Chemotherapy	Completed CT Cycles	Pelvic EBRT	VBT	Paraaortic Evaluation
P1	58	Endometrioid carcinoma	2	Yes	3.5	Yes	6	Yes	No	No
P2	61	Endometrioid carcinoma	3	Yes	5.0	Yes	6	Yes	Yes	Yes
P3	54	Endometrioid carcinoma	2	No	2.8	Yes	6	Yes	No	No
P4	49	Endometrioid carcinoma	1	No	2.2	Yes	6	No	Yes	No
P5	64	Serous carcinoma	3	Yes	4.8	Yes	6	Yes	No	Yes
P6	52	Endometrioid carcinoma	2	No	3.0	Yes	6	Yes	No	No
P7	57	Endometrioid carcinoma	2	Yes	3.6	Yes	6	Yes	No	No
P8	46	Endometrioid carcinoma	1	No	2.0	Yes	4	No	Yes	No
P9	69	Endometrioid carcinoma	3	Yes	6.0	Yes	6	Yes	No	No
P10	55	Endometrioid carcinoma	2	No	3.1	No	0	No	No	No

CT: chemotherapy; EBRT: external beam radiotherapy; LVSI: lymphovascular space invasion; SLN: sentinel lymph node; VBT: vaginal brachytherapy.

## Data Availability

The datasets generated and/or analyzed during the current study are not publicly available due to institutional data protection policies but are available from the corresponding author (B.C.) on reasonable request and with permission of the institutional review board.

## Supplementary Materials

The following supporting information can be downloaded at https://www.mdpi.com/article/10.3390/medicina62010043/s1, Video S1: Retroperitoneal vNOTES access, pelvic sentinel lymph node mapping, and vNOTES-assisted hysterectomy with bilateral salpingo oophorectomy in a patient with presumed early-stage endometrial cancer; Table S1: Sentinel lymph node (SLN) mapping algorithm applied during retroperitoneal vNOTES staging for presumed early-stage endometrial cancer.

## Abbreviations

BMI	Body mass index
BSO	Bilateral salpingo-oophorectomy
CT	Computed tomography
EC	Endometrial cancer
ECOG	Eastern Cooperative Oncology Group
EIN	Endometrial intraepithelial neoplasia
EBRT	External beam radiotherapy
FDG	Fluorodeoxyglucose
FIGO	International Federation of Gynecology and Obstetrics
ICG	Indocyanine green
LN	Lymph node
LVSI	Lymphovascular space invasion
MIS	Minimally invasive surgery
MRI	Magnetic resonance imaging
PET/CT	Positron emission tomography/computed tomography
SLN	Sentinel lymph node
VAS	Visual analog scale
vNOTES	Vaginal natural orifice transluminal endoscopic surgery
VBT	Vaginal brachytherapy
