# Supplementary material for: Does Retroperitoneal vNOTES Sentinel Lymph Node Mapping Represent a Feasible Staging Option in Presumed Early-Stage Endometrial Cancer?
Source: Medicina (Kaunas). 2025 Dec 25;62(1):43. doi: 10.3390/medicina62010043 (PMC12843302; doi:10.3390/medicina62010043)
Supplement: Supplementary file 1 [file medicina-62-00043-s001.zip › medicina-4031629-supplementary/medicina-4031629-supplementary-table s1.pdf]

Supplementary Table S1. Sentinel lymph node (SLN) mapping algorithm applied during retroperitoneal vNOTES staging for presumed early-stage endometrial cancer

Intraoperative SLN mapping outcome	Surgical management strategy
Successful bilateral SLN mapping	Excision of all mapped sentinel lymph nodes and any macroscopically suspicious pelvic nodes
Unilateral SLN mapping	Excision of mapped SLN on the detected side combined with side-specific pelvic lymphadenectomy (external iliac, internal iliac, and obturator regions) on the unmapped hemipelvis
Complete SLN mapping failure	Pelvic lymphadenectomy performed at the surgeon's discretion based on intraoperative assessment
Suspicious or enlarged lymph nodes (regardless of mapping status)	Mandatory excision

The sentinel lymph node algorithm was applied in accordance with contemporary guideline-based recommendations for endometrial cancer staging.
